# Where Do Our Children Go? Understanding the Impact of Institutionalization on Emotion Regulation, Attention, and Sleep

**DOI:** 10.3390/children12040448

**Published:** 2025-03-31

**Authors:** Sandra Figueiredo, Patrícia Silvestre

**Affiliations:** Psychology Research Centre (CIP and University Research Centre in Psychology (CUIP)), Department of Psychology, Universidade Autónoma de Lisboa Luís de Camões, Palácio Dos Condes Do Redondo, R. de Santa Marta 56, 1169-023 Lisbon, Portugal; patriciasilvestre14@hotmail.com

**Keywords:** institutionalization, emotional regulation, attention, sleep routines, childhood care

## Abstract

Background/Objectives: Emotional regulation and attention are markedly underdeveloped in institutionalized children (IC) relative to non-institutionalized children (NIC). Caregivers in temporary institutional settings tend to exhibit contingency-responsive behaviors with limited affective engagement, which may restrict optimal socio-emotional development. Despite the critical role that sleep routines play in child development, their association with emotional regulation and attention in the context of institutionalization remains insufficiently explored. The present study aimed to assess the impact of institutionalization on emotional regulation, attention, and sleep hygiene in childhood, as well as to investigate whether sleep mediates the relationship between emotional regulation and attention. Methods: A total of 110 children aged 7 to 11 years (N = 55 IC; N = 55 NIC) were assessed using the Emotion Regulation Checklist (ERC), the Cancellation Test (Toulouse-Piéron), and the Children’s Chronotype Questionnaire. Results: Regression analyses and parametric tests revealed significant differences between IC and NIC groups. IC exhibited lower emotional regulation (β = 8.018, *p* < 0.05) and poorer attention (β = 3.818, *p* < 0.05) compared with NIC. Additionally, the MidSleep Point (MSP) was significantly different between groups, with NIC demonstrating shorter sleep periods (β = −1158.545, *p* < 0.05), contrary to expectations. Conclusions: These findings suggest that prolonged institutionalization impairs socio-emotional development, with downstream effects on cognitive functioning, particularly attention. While differences in sleep routines between IC and NIC were observed, sleep did not appear to directly mediate the relationship between emotional regulation and attention, nor did it present a primary risk factor compared with the broader developmental challenges associated with institutional care.

## 1. Introduction

Globally, up to 8 million children live in temporary institutional settings, a number exacerbated by ongoing waves of refugee displacement [1,2]. Institutionalization presents significant challenges to emotional, cognitive, and behavioral development [3,4]. Despite growing research on childhood development, the specific effects of institutionalization remain poorly understood, particularly in relation to the dynamic interactions among emotion regulation, attention, and sleep. Emerging evidence suggests that effective emotion regulation in preschool-aged children supports adaptive behaviors, including social engagement and cooperative play. However, these findings primarily pertain to children raised in biological family settings, where early play experiences within the home environment serve as crucial developmental foundations [5,6]. Co-development between parents and young children is generally beneficial for fostering emotion regulation skills [5,7].

In contrast, institutionalized children (IC) often lack the buffering effects of these interactions, particularly in settings where high child-to-caregiver ratios impose constraints on individualized attention and necessitate rigid routines [6]. Childhood constitutes a critical period for socio-emotional and cognitive development, during which institutionalization can significantly disrupt these processes. Frequent turnover among institutional caregivers—reportedly as high as 60 to 100 times per year—exacerbates attachment instability, resulting in an unpredictable and inconsistent caregiving environment [8,9]. Research consistently shows that institutional care environments provide insufficient emotional and social support, negatively affecting emotion regulation and executive functions, particularly attention [10,11,12].

Emotion regulation refers to the ability to identify, process, and adaptively express emotions, which is foundational to socio-emotional development [13]. Typically, this developmental trajectory begins within the family unit, where parents serve as primary models for children’s self-regulation [14,15]. In contrast, institutionalized children (IC) often experience disruptions in familial structures and frequent transitions between institutional settings or foster care, often within short timeframes. These unstable conditions impede the development of adaptive emotional regulation, placing IC at a disadvantage compared with their non-institutionalized peers (NIC). As a result, IC exhibit heightened emotional reactivity, increased negative behaviors, and deficits in cognitive functions such as attention [4,6,16].

Attention, a key cognitive skill, is notably vulnerable to the effects of institutionalization due to the lack of adequate environmental stimulation in care facilities [4,17]. Attention plays a fundamental role in learning by enabling the encoding of relevant environmental stimuli, which serve as the basis for cognitive and behavioral development. Efficient attentional processes are also directly linked to academic achievement. Empirical research indicates that institutionalized children face an elevated risk of academic and behavioral difficulties compared with their non-institutionalized peers [3,18,19]. Beyond its cognitive function, attention is also influenced by sleep quality, a crucial yet understudied factor in the context of institutional care. Poor sleep hygiene has been linked to deficits in attention and diminished academic performance, particularly in tasks requiring fluid intelligence [16]. Both wakefulness and vigilance are shaped by endogenous mechanisms as well as external influences, including social schedules, which tend to be rigidly structured in institutional settings [20,21]. Sleep routines among institutionalized children (IC) differ markedly from those of their non-institutionalized counterparts (NIC) due to communal sleeping arrangements and regimented schedules, in contrast with the familial co-sleeping practices often observed in NIC households [19]. Disruptions in sleep during childhood have been associated with impairments in attention, memory, and emotion regulation, underscoring the essential role of sleep in developmental trajectories [22,23,24].

Despite the well-documented developmental challenges faced by institutionalized children (IC), the specific relationships among sleep behavior, emotion regulation, and attention in this population remain largely unexplored. Existing research on the association between sleep and developmental outcomes has predominantly focused on institutionalized adults rather than children [25,26,27]. Gaining a deeper understanding of sleep patterns and their developmental implications in young IC is critical for identifying targeted interventions aimed at mitigating the adverse effects of institutionalization.

This study aims to address this gap by proposing and testing a conceptual model that examines the relationships between institutionalization, emotion regulation, attention, and sleep routines in young children. The practical implications of these findings will be discussed in the context of childhood assessments and interventions.

## 2. Method

Drawing on the reviewed literature, we hypothesize the following relationships between attention, emotion regulation, and sleep routines in young children:

Within the context of institutionalization, preschool-aged children are anticipated to exhibit attention deficits linked to reduced emotion regulation capacities.

**Hypothesis** **1.**  *Children in institutional care are expected to exhibit attention deficits, which are strongly associated with reduced capacities for emotion regulation.*

**Hypothesis** **2.**  *Institutionalized children (IC) are anticipated to employ maladaptive strategies for emotion regulation, in comparison with NIC, resulting in a poorer emotional adjustment compared with their non-institutionalized peers.*

**Hypothesis** **3.**  *Maladaptive emotion regulation strategies in institutionalized children (IC) are hypothesized to be strongly correlated with diminished attention performance, suggesting an interdependence between emotional regulation and cognitive attention processes.*

**Hypothesis** **4.**  *Distinct sleep and vigilance patterns are expected between institutionalized children (IC) and non-institutionalized children (NIC), with these differences having corresponding effects on attention and emotion regulation.*

**Hypothesis** **5.**  *Institutionalization is expected to contribute to a lower MidSleep Point in institutionalized children (IC), which in turn may influence both their regulatory processes and attentional performance.*

### Participants

This study included 55 children institutionalized (IC) and 55 children non-institutionalized (NIC), aged between 7 and 11 years old, where 56 (50.9%) were male and 54 (49.1%) were female. IC resided in temporary institutions, NIC lived with their biologic parents, with both samples referring to Portugal. Further, 91 (82.7%) were Portuguese, 5 (4.5%) were from Angola, 3 (2.7%) were born in Guinea, and 2 (1.8%) referred to Chinese and Brazilian nationalities. Regarding the chronotype, 13 (11.8%) were morning type, 66 (60%) identified as intermediate, and 31 (28.2%) were evening type.

Inclusion criteria: (i) preschool age; (ii) both IC and NIC attending primary and 2nd cycle of school, public, or private; and (iii) with at least 6 months of residence in institutions.

Exclusion criteria: (i) no mental illness diagnosed and (ii) attending or attended psychiatric therapy.

Selection of participants was convenient and previous informed consent of parents (NIC) and institutions (IC) had been obtained.

## 3. Instruments

A sociodemographic questionnaire was provided to the parents and institutions. They completed the questionnaire pertaining to the age, sex, preschool year, nationality, profession of father and mother, and number of siblings (living in household). Additionally, IC answered (institutions answered) about the motive for institutionalization and duration of institutionalization.

Emotion Regulation Checklist (ERC): Portuguese version [28] of ERC from Shields and Cicchetti [13] assessed the emotion regulation regarding intensity and valence of emotions, including adaptive and maladaptive strategies. The 24-item scale measures emotion regulation through two scales: emotion regulation with α = 0.96 (emotional expressions adequacy) and lability/negativity with α = 0.83 (negative intensity, anger, reactivity, and alterations of humor). Only adults could answer ERC and within the proximity of the child being evaluated. ERC presents a 4-point Likert Scale: 1: never, 2: sometimes, 3: many times, and [4] 4: almost always. Parents (for NIC) and institutions (for IC) answered the test. For the Lability/Negativity scale, Cronbach’s alpha values of 0.96 were reported, while for the Emotional Regulation scale, the Cronbach’s alpha was 0.83.

Cancellation Test from BANC [29]: Portuguese version adapted, in the context of Neuropsychological Assessment Battery of Coimbra (BANC), from the Toulouse-Piéron Cancellation Test. This test measures the selective and sustained attention, based on speed scores related to the number of correct identifications. Young children answered the test under the supervision of the research team because of the need for concentration during the task.

This test has two versions, which are administered according to the age of the child/adolescent being tested. One version is intended for children aged 5 to 9 years and contains 2 symbols, while the other version is for children aged 10 to 15 years and includes 3 symbols. The test takes approximately 10 min to complete and is filled out individually by the children/adolescents in the presence of the researcher, so the presence of a professional in the field is not required [29]. At the top of the test sheet, there are 2 or 3 symbols (depending on the version, as mentioned) that the child/adolescent must find among the 1000 symbols in the remaining lines (i.e., there are 25 lines in total, each containing 40 symbols). All of the symbols are identical, but have slight differences (i.e., external lines in different directions). The test is scored based on the total number of errors (E), correct responses (A), and omissions (O), with the total score (TS) being calculated from these three factors [29]. The maximum score for cancelling 2 symbols is 25 points, and for cancelling 3 symbols, it is 37.5 points. By comparing these scores with standardized norms for the participant’s age, an assessment of sustained and selective attention can be completed.

Children’s Chronotype Questionnaire (CCTQ): Portuguese version of CCTQ [30], based on the original version of Werner et al. [31], was administered to evaluate the sleep routines and the chronotype (diurnal preferences of children regarding activities and sleep). The chronotype is divided into three: morningness, intermediate, and eveningness. This instrument presents 27 items distributed across three scales: (i) MidSleep Point or MidSleep Phase (MSP or MSF) with 16 items, measured by the total of hours and minutes considering different sleep behaviors (example: bedtime, time of feeling sleepy, awake time) in school days (weekdays) and in free days (weekends). (ii) Morningness/Eveningness (M/E): 10 items addressing sleep preferences considering different activities of vigilance and sleep in specific schedules. The punctuation can vary from 10 to 49. (iii) Chronotype: item 27 assesses the children’s chronotype from the parents’ perspective (α = 0.71). CCTQ is a self-report questionnaire answered by parents or another tutor of the child, in the case of NIC.

## 4. Procedure

The study received approval from the Ethics Committee of the Department of Psychology at Universidade Autónoma de Lisboa (UAL) at [University Name]. Participants, including institutionalized children (IC) and non-institutionalized children (NIC), were recruited through convenience sampling from Portuguese institutions and families who provided informed assent, respectively. Permission to use the instruments was obtained by their authors, who consented to the replication of their tools for this study. Both participant groups were closely monitored throughout the study’s presentation and participation process. Consent was secured from parents and institutional heads for children’s enrolment, with the option for guardians to withdraw their children at any stage. Over a three-month period (March to May 2022), two trained researchers (*N* = 2) oversaw the administration of four assessment instruments, as well as the subsequent data analysis and interpretation. Investigators were present to administer the ERC and the cancellation test during different session in order to the attend IC and NIC samples. The sleep questionnaire was completed by the caregivers for IC, and by parents for NIC. Data collection adhered to strict anonymity protocols at all times, ensuring the confidentiality of the participant information.

## 5. Analysis

Regarding the assumptions of simple linear regression, specifically the linearity of the relationship between the independent variables and the dependent variable (graphical analysis), independent of residuals (Durbin-Watson test), normality of residuals (Kolmogorov-Smirnov test), and homogeneity of variances (graphical analysis), these were assessed and were generally satisfied. For samples larger than 30, normality of distribution was assumed based on the central limit theorem. Additionally, homogeneity of variances was examined using Levene’s test, with the significance level for rejecting the null hypothesis set at α < 0.05. Regarding the Chi-square assumption that no more than 20% of the cells should have expected frequencies below 5, this was analyzed. In cases where this assumption was not met, the Chi-square test with Monte Carlo simulation was employed. Differences were analyzed using standardized adjusted residuals. The groups were equivalent in terms of age, *t*(108) = 1.339, *p* = 0.184, and gender, Fisher’s exact test, *p* = 0.567. However, in the group of institutionalized children, the proportion of grade retentions was significantly higher, as indicated by Fisher’s exact test, *p* = 0.001.

Pearson’s correlations and multiple linear regressions were employed to examine the relationships between emotion regulation, sleep, and attention. To ensure the homogeneity of variance across the samples, the Durbin-Watson and Kolmogorov-Smirnov tests were applied during the regression analysis. Additionally, Student’s *t*-test and Fisher’s exact test were conducted on independent samples to compare the means between institutionalized children (IC) and non-institutionalized children (NIC). Specifically, the MidSleep Point (MSP) was calculated in accordance with the method outlined by Roenneberg et al. [32].

We conducted a power analysis to determine the adequacy of the sample size, utilizing frequency distributions, visual binning, and data validation procedures. All cases were verified and confirmed as valid. Regarding the visual binning and descriptive frequency analysis, the statistics indicated only three to four missing cases across the two instrument scales. These missing cases did not impact the sample or the data analysis, as confirmed by the data validation tests. The verification of variables (data validation) further confirmed that the sample size was adequate, with no detected outliers (approximating *N* = 110).

## 6. Results

### 6.1. Attention and Institutionalization

Regarding attention, Student’s *t*-test on the independent samples revealed differences (*t*(108) = −3.45, *p* < 0.01) between IC and NIC, with prejudice for IC who showed a lower performance in the cancellation test. NIC were punctuated higher in the attention test (M = 82.3; SD = 10.4) compared with IC (M = 74.1; SD = 12.7).

The simple linear regression model with the variable “being institutionalized or not” as the independent variable and the performance on the symbol detection test as the dependent variable explained 22.5% of the total variance of the latter variable, and was statistically significant: F(1, 108) = 31.736, *p* < 0.001. The variable “being institutionalized or not” proved to be a significant predictor of performance on the symbol detection test (B = 3.818, *p* < 0.001).

### 6.2. Emotional Regulation and Institutionalization

NIC scored higher in emotional regulation adequacy (M = 36.36 for NIC vs. 22.34 for IC), with statistically significant difference (*t*(108) = 13.074, *p* < 0.001), while IC scored significantly higher in lability/negativity (36.52 vs. 18.64), *t*(107) = 11.456, *p* < 0.001.

The regression model showed that being institutionalized explained 61.3% of the total variance for emotional regulation: F(1, 108) = 170.909, *p* < 0.001, demonstrating a high predictive value for emotion regulation in both dimensions (B = 8.018, *p* < 0.001). As the regression coefficient was positive, non-institutionalized children obtained significantly higher scores in emotional regulation. Specifically, for lability/negativity dimensions, being institutionalized explained, in a statistically significant manner, 55.1% of the total variance (F(1, 107) = 131.244, *p* < 0.001). As the regression coefficient was negative (B = −14.879, *p* < 0.001), non-institutionalized children scored lower than institutionalized children in lability/negativity (see Table 1).

### 6.3. Emotion Regulation, Attention and Institutionalization

A negative and significant correlation was found between lability/negativity and attention (r = −0.288, *p* = 0.033) in institutionalized children (IC). Thus, the higher the lability/negativity, the greater the attention difficulties. In contrast, this correlation was not significant in non-institutionalized children.

### 6.4. Sleep Preferences and Institutionalization

Regarding sleep preferences, in terms of social timing, the differences between institutionalized and non-institutionalized children were all statistically significant (*p* < 0.001), with institutionalized children waking up earlier, getting up earlier, going to bed earlier, but taking longer to fall asleep. Specifically, IC had earlier times for all sleep habits compared with NIC. However, on the contrary, NIC had more difficulty (more time experiencing) before asleep after going to bed. Also, NIC had more sleepiness during the day (see Table 2).

Institutionalization explained 6.9% of the total variance toward “Corrected MidSleep Point” or MSP (F (1, 108) = 7.981, *p* < 0.001). The variable “being institutionalized or not” proved to be a significant predictor of the MidSleep PPoint (B = −1158.545, *p* < 0.001). The regression result was negative, thus non-institutionalized children had a significantly lower MSP.

Regarding sleep preferences and specifically concerning the chronotype, IC and NIC did not differ in a significant manner, despite a higher proportion in NIC for eveningness compared with IC.

Additionally, emotion regulation was revealed to be significant predictor of attention performance, with a β-coefficient of 0.32 (*p* < 0.01), explaining 10% of the observed variance (R^2^ = 0.10). Sleep habits also proved to be predictive for attention and emotion regulation among IC, although to a lesser extent, with β = 0.25 (*p* < 0.05) and R^2^ = 0.06.

In summary, both emotion regulation—across the dimensions of lability and negativity—and attention were more significantly impaired in institutionalized children (IC). The institutionalized condition was found to be negatively correlated with emotion regulation and attention. However, sleep preferences did not exhibit a strong direct effect on either attention or emotion regulation. Notably, sleep behaviors on school days differed between IC and non-institutionalized children (NIC), with IC children showing a propensity for earlier sleep schedules. In conclusion, emotion regulation strategies were found to have a more pronounced influence on attention than the reverse, particularly in institutionalized children.

## 7. Discussion

Regarding the conditions considered in the analysis of the present study, it was confirmed that preschool-aged children in institutional settings exhibited attention difficulties linked to low levels of positive emotional regulation. Maladaptive and negative emotional regulation strategies were also observed. Moreover, it was found that this maladaptive regulation was significantly associated with attention deficits. A distinction between institutionalized children (IC) and non-institutionalized children (NIC) in terms of sleep habits was established, although the relationship between these different sleep patterns and attention and emotional regulation is not strong. Additionally, it was confirmed that the MidSleep Point (MSP) was lower in IC compared with NIC.

The data confirmed a negative relationship between attention and negative emotional regulation in institutionalized children: the greater the reliance on negative emotional regulation, the lower the level of attention. Furthermore, IC and NIC demonstrated distinct sleep habits, such as differences in wake-up and bedtimes, with IC sleeping less, as indicated by the lower MSP and greater difficulty falling asleep after bedtime. No significant differences in chronotype were observed between the two groups. These findings help explain the low predictive power of the “sleep” variable in relation to emotional regulation and attention in both IC and NIC, particularly among IC. Institutionalization emerged as a predictor of variance in sleep behavior, with emotional regulation in IC and NIC serving as a predictor of attention in both groups.

Thus, the results of this study confirm that institutionalization has a significant negative relationship with children’s emotional and cognitive development, supporting previous studies [3,4]. Institutionalized children demonstrated a poorer performance in attention and emotional regulation, likely due to the lack of consistent social and emotional stimuli in institutional environments [8,33]. Regarding attention specifically, our results are consistent with the literature, where authors have mentioned that children living in institutional care settings exhibit more cognitive deficits than those living in their natural environment, particularly in attention capacity [27,33,34]. In the same vein, the findings align with the pilot study conducted by De Luccia-Rivaben and Fiamenghi [35], in which the authors stated that institutionalization may have a negative effect on children’s intellectual development. These data are particularly concerning when considering the study by Wade et al. [36], which concluded that the children in the sample, even after leaving the institution where they had been sheltered, exhibited significant deficits in cognitive skills, particularly in language, attention, memory, and executive function.

### 7.1. Attention and Emotional Regulation

The strong relationship between emotional regulation and attention suggests that the ability to manage emotions plays a key role in children’s cognitive performance, particularly attention. This supports earlier research indicating that children who struggle more with emotional regulation, as seen in the institutionalized group, tend to face challenges in concentration and executive function [36]. Existing literature on institutionalization highlights that less adaptive emotional regulation strategies, such as avoidance or suppression (both emotional and expressive), are common among children who experience early adversity, such as being institutionalized and separated from their biological families [37,38].

The findings of this study align with those of Cruz et al. [6], which showed that children exposed to inconsistent care in institutional settings experienced greater difficulties in emotional regulation, negatively impacting academic performance and social relationships. Regarding social relationships, IC exhibited higher emotional reactivity and negative emotions when facing events, which is supported by recent studies on IC and disruptive emotions [8,16].

It is also important to highlight the relationship between cognitive development and emotional regulation, which is connected to previous variables. Thus, in the case of non-institutionalized children, where conditions are favorable for normal emotional development, they also exhibited good cognitive abilities; however, in the case of institutionalized children, given the adversities and obstacles to healthy emotional development, this tends to be reflected in cognitive difficulties as well [35,38].

### 7.2. Impact of Sleep Routines and Sleep Hygiene

Although institutionalized children (IC) exhibited stricter sleep routines, such as earlier bedtimes and wake-up times, the results indicate an emotionally risky quality within the institutional environment [6]. Despite early sleeping and waking schedules, IC reported greater difficulty falling asleep, which reduced the total sleep duration and consequently lowered their MidSleep Point (MSP). This helps explain why regression analysis showed that sleep was not a predictor of emotional regulation and attention when differentiating IC from non-institutionalized children (NIC). These findings align with recent studies that suggest emotional deprivation or reduced cognitive stimulation in care institutions can undermine the effectiveness of sleep routines, even when sleep hygiene practices are followed [3,5,18,24,39]. Research by Cadima et al. [7] and Figueiredo [18] highlights the close relationship between sleep habits and children’s academic performance and emotional well-being. Children who maintain good sleep hygiene practices tend to have better emotional regulation and attention, which translates into a more positive cognitive performance. In the context of institutionalization, however, this relationship was not observed in the present study, pointing to a disadvantage in executive functioning during childhood [12,22].

Based on the results obtained here, we can verify that they do not fully align with the existing literature in this field. The formulated hypothesis was rejected, as statistically significant differences were found between the two groups in all of the analyzed components. Specifically, it was observed that institutionalized children wake up earlier, get up earlier, and go to bed earlier, although they take longer to fall asleep [40,41]. Institutions are responsible for promoting and ensuring the well-being of the children in their care, and it is expected that there are strict and well-defined sleep routines—which can be understood as wake-up times, get-up times, and bedtimes that are earlier in institutions than in natural family homes—to ensure that children obtain the necessary amount of sleep for their age and normal development [40,41].

Therefore, promoting healthy sleep habits is essential, especially in institutional environments, where children are less likely to receive adequate emotional support due to staff turnover and the nature of the attachment formed with caregivers [7,9,10,42].

## 8. Clinical and Educational Implications

The findings of this study carry significant implications for public policy and intervention strategies. Implementing programs designed to promote emotional regulation and cognitive development in institutionalized children could help mitigate some of the adverse effects of institutionalization. For instance, mindfulness-based interventions have been shown to effectively improve executive function and emotional regulation in children from vulnerable backgrounds [43,44].

Furthermore, it is crucial for care institutions to adopt practices that cultivate a safe emotional environment, supporting children’s well-being while promoting the development of healthy sleep habits. This could involve training caregivers on the importance of emotional regulation and sleep hygiene, as well as establishing routines that ensure a stable and predictable environment [45,46].

Considering care institutions hosting children, particularly those classified as temporary placements, it is crucial for caregivers to adopt best practices that prioritize children’s sleep schedules, both within the institutions and during their external activities, such as school. It is essential to assess potential asynchrony between these schedules and make necessary adjustments. Such practices could directly influence the improvement of emotional regulation and optimize attention.

Regarding attention, children raised in institutional care may be more susceptible to developing attention-related difficulties during their development. Therefore, caregivers should consistently monitor these children using assessment tools to evaluate attention, while also considering its connection to emotional regulation. This holistic approach would help caregivers better understand and address the children’s needs.

## 9. Conclusions

This study presents strong evidence of the adverse effects of institutionalization on children’s emotional and cognitive development, emphasizing significant challenges in emotional regulation and attention. Statistical analyses, supported by existing literature, highlight that while stricter sleep routines within institutional settings may offer limited benefits, fostering ongoing emotional development and providing consistent support are critical for alleviating the negative impacts of institutional care.

Future research should prioritize investigating interventions designed (as longitudinal) to enhance adaptive emotional regulation strategies and ensure more reliable socio-emotional support for institutionalized children. Further comprehensive data analyzes are important for future studies on this topic. Moreover, the link between sleep routines and emotional development deserves further exploration, particularly in light of environmental factors influencing sleep hygiene practices [24,39,46].

### Limitations of the Study

It is important to mention that this study has some limitations, particularly in terms of the generalization of its conclusions, as the sample is not representative of the entire institutionalized population. Therefore, the conclusions drawn apply solely to this study.

Additionally, the discrepancies in terms of the reasons/motives for institutional placement, how long the children have been in care, or the opportunity for contact with their family of origin constitute another limitation of the study. However, these discrepancies could not be controlled, as the sample was not selected with the intention of being homogeneous in the mentioned parameters.

As suggestions for future research, it would be relevant to further explore the relationship between chronotype and morning−evening preference in other similar samples, as well as to examine whether there is a mediation effect of sex/gender on the aforementioned tendency. It would also be valuable to assess other areas of cognitive skills, such as memory and reasoning, using psychometric tests, as these abilities are frequently noted in the literature as being deficient in institutionalized populations. Additionally, a follow-up study could be conducted to determine whether the differences remain significant over time or if age mitigates them.

## Figures and Tables

**Table 1:** IC and emotion regulation.

Model	Non-Standardized Coef. Std.		Standardized Coef, Beta	*t*	*p*
	B	Error			
1 (Constant)	22,345	0.434		51,526	0.000
IC	8018	0.613	0.783	13,074	0.000 ***
	33,527	0.914		36,676	0.000
IC	−14,879	1.299	−0.742	−11,456	0.000 ***

* *p* ≤ 0.05, ** *p* ≤ 0.01, *** *p* ≤ 0.001; Coef. = coefficients values.

**Table 2:** Habits of sleep in the night before school days.

	Institutionalized		Non Institutionalized		
	M	SD	M	SD	*p*
School-days—wake-up time	7:03	0:27	7:26	0:25	0.001 ***
School-days—getting-up time	7:11	0:24	7:31	0:23	0.001 ***
School days—full awake time	7:19	0:24	7:38	0:23	0.001 ***
School days—feeling sleep	21:00	0:24	21:35	0:31	0.001 ***
School days—going to bed	21:13	0:28	21:48	0:29	0.001 ***

School days, fall asleep; M, mean; SD, Standard Deviation; * *p* ≤ 0.05, ** *p* ≤ 0.01, *** *p* ≤ 0.001.

## Data Availability

Data generated and analyzed during this study are included and clearly identified in this article.

## References

[1] Human Rights Watch (2017). Children with Disabilities: Deprivation of Liberty in the Name of Care and Treatment. Prot. Child. Against Torture Deten. Glob. Solut..

[2] Human Rights Watch (2021). Connections Between Children’s Institutional Care and Education. Human Rights Watch. https://www.hrw.org/news/2021/10/22/connections-between-childrens-institutional-care-and-education.

[3] Chen F. (2020). Co-development of emotion regulation: Shifting from self-focused to child-focused *perezhivanie* in everyday parent-toddler dramatic collisions. Early Child Dev. Care.

[4] Hamamcı B., Balaban Dagal A. (2021). Self-regulation and play: How children’s play directed with executive function and emotion regulation. Early Child Dev. Care.

[5] Kaya E., Günaydin N. (2023). Examination of preschool children’s anxiety with a mediator model: The effect of mother’s emotion regulation skills and child’s emotion expression styles. Early Child Dev. Care.

[6] Cruz S., Sousa M., Mateus V. (2024). Emotion Regulation and Cognitive and Social Functioning in Early Development: The Interface between Neurophysiological and Behavioural Perspectives. Emotional Intelligence-Understanding, Influencing, and Utilizing Emotions.

[7] Cadima J., Ferreira T., Guedes C., Vieira J., Leal T., Matos P. (2016). Risk and emotion regulation in pre-schoolers: The quality of interactions among caregivers as potential mediator [Risco e regulação emocional em idade pré-escolar: A qualidade das interações dos educadores de infância como potencial moderador]. Análise Psicológica.

[8] McCall R., Groark C., Hawk B., Julian M., Merz E., Rosas J., Muhamedrahimov R., Palmov O., Nikiforova N. (2019). Early Caregiver-Child Interaction and Children’s Development: Lessons from the St. Petersburg-USA Orphanage Intervention Research Project. Clin. Child Fam Psychol. Rev..

[9] Vorkapic T., Velan D. (2023). The importance of positive emotions among early childhood educators. Teach. Dev..

[10] Batki A. (2017). The impact of early institutional care on emotion regulation: Studying the play narratives of post-institutionalized and early adopted children. Early Child Dev. Care.

[11] Zeanah C.H., Egger H.L., Smyke A.T., Nelson C.A., Fox N.A., Marshall P.J., Guthrie D. (2009). Institutional rearing and psychiatric disorders in Romanian preschool children. Am. J. Psychiatry.

[12] Lollies F., Schnatschmidt M., Bihlmeier I., Genuneit J., In-Albnon T., Holtmann M., Legenbauer T., Schlarb A.A. (2022). Associations of sleep and emotion regulation processes in childhood and adolescence—A systematic review, report of methodological challenges and future directions. Sleep Sci..

[13] Shields A., Cicchetti D. (1997). Emotion regulation among school-age children: The development and validation of a new criterion Q-sort scale. Dev. Psychol..

[14] Montealegre-Ramón M.d.P., Martínez-Fuentes M.T., Pérez-López J., Sierra-García P. (2024). Maternal sensitivity, early interactions and infant attachment: A systematic review. Early Child Dev. Care.

[15] Rodcharoen P., Neuhauser A., Kalkusch I., Schaub S., Lanfranchi A., Klaver P., Oeri N. (2024). Behavioral self-regulation development in at-risk families: The role of family resources. Early Child Dev. Care.

[16] Sherr L., Roberts K.J., Gandhi N. (2017). Child violence experiences in institutionalised/orphanage care. Psychol. Health Med..

[17] Troller-Renfree S., Nelson C., Zeanah C., Fox N. (2016). Deficits in error monitoringare associated with externalizing but not internalizing behaviors among children with a history of institutionalization. J. Child Psychol. Psychiatry.

[18] Figueiredo S., Hipólito J. (2022). Association between parents’ supervision and the sleep habits of children: The impact of educational background of families in balanced sleep and wakefulness. Biol. Rhythm. Res..

[19] Larose M.P., Côté S.M., Ouellet-Morin I., Maughan B., Barker E.D. (2021). Promoting better functioning among children exposed to high levels of family adversity: The protective role of childcare attendance. J. Child Psychol. Psychiatry.

[20] Isenhour J., Raby K.L., Dozier M. (2021). The persistent associations between early institutional care and diurnal cortisol outcomes among children adopted internationally. Dev. Psychobiol..

[21] Stenson A.R., Kurinec C.A., Hinson J.M., Whitney P., Van Dongen H.P. (2021). Total sleep deprivation reduces top-down regulation of emotion without altering bottom-up affective processing. PLoS ONE.

[22] Bernier A., Beauchamp M.H., Bouvette T.A., Carlson S.M., Carrier J. (2013). Sleep and Cognition in Preschool Years: Specific Links to Executive Functioning. Child Dev..

[23] Figueiredo S. (2024). Chronotypes, Disruptive Behaviour, and Schedules in Classrooms: ‘Morningness’ and Psychomotor Agitation. CEPS J. Cent. Educ. Policy Stud. J..

[24] Hysing M., Heradstveit O., Harvey A.G., Nilsen S.A., Bøe T., Sivertsen B. (2022). Sleep problems among adolescents within child and adolescent mental health services. An epidemiological study with registry linkage. Eur. Child Adolesc. Psychiatry.

[25] Leiza J.G., Garcia M.A., De Llano J.A., Martinez D.M., De La Higuera P.C., Lopez C.A. (2013). Influence of institutionalization on the sleep pattern in elderly population. Sleep Med..

[26] Marengoni A., Tazzeo C., Calderón-Larrañaga A., Roso-Llorach A., Onder G., Zucchelli A., Vetrano D.L. (2021). Multimorbidity patterns and 6-year risk of institutionalization in older persons: The role of social formal and informal care. J. Am. Med. Dir. Assoc..

[27] Martins da Silva R., Afonso P., Fonseca M., Teodoro T. (2020). Comparing sleep quality in institutionalized and non-institutionalized elderly individuals. Aging Ment. Health.

[28] Melo A. (2005). Emotions in School Period: Parental Strategies Toward Emotional Expression and Symptomatology of Internalization and Externalization in Children. [Emoções no Período Escolar: Estratégias Parentais Face à Expressão Emocional e Sintomas de Internalização e Externalização da Criança]. Master’s Thesis.

[29] Simões M., Albuquerque C., Salomé Pinho M., Vilar M., Pereira M., Lopes A., Santos M.J.S., Alberto I., Lopes C., Martins C. (2016). Neuropsychological Evaluation Battery of COIMBRA (Banc). [Bateria de Avaliação Neuropsicológica de COIMBRA (BANC), (1st ed.)] Cegoc. https://psycnet.apa.org/doiLanding?doi=10.1037/t66286-000.

[30] Couto D., Allen Gomes A., Pinto de Azevedo M.H., Bos SM C., Leitão J.A., Silva C.F. (2014). The European Portuguese version of the Children ChronoType Questionnaire (CCTQ): Reliability and raw scores in a large continentalsample. J. Sleep Res..

[31] Werner H., LeBourgeois M.K., Geiger A., Jenni O.G. (2009). Assessment of chronotype in four-to eleven-year-old children: Reliability and validity of the Children’s Chronotype Questionnaire (CCTQ). Chronobiol. Int..

[32] Roenneberg T., Kuehnle T., Pramstaller P.P., Ricken J., Havel M., Guth A., Merrow M. (2004). A marker for the end of adolescence. Curr. Biol..

[33] Zeanah C.H., Humphreys K.L., Fox N.A., Nelson C.A. (2017). Alternatives for abandoned children: Insights from the Bucharest Early Intervention Project. Curr. Opin. Psychol..

[34] Hodel A.S., Hunt R.H., Cowell R.A., Van Den Heuvel S.E., Gunnar M.R., Thomas K.M. (2015). Duration of early adversity and structural brain development in postinstitutionalized adolescents. NeuroImage.

[35] De Luccia-Rivaben M., Fiamenghi G. (2014). Comparison between institutionalized and non-institutionalized children’s intellectual level: Pilot study. Encontro Rev. Psicol..

[36] Wade M., Carroll D., Fox N.A., Zeanah C.H., Nelson C.A. (2022). Associations between Early Psychosocial Deprivation, Cognitive and Psychiatric Morbidity, and Risk-taking Behavior in Adolescence. J. Clin. Child Adolesc. Psychol..

[37] McLaughlin K.A., Prinstein M. (2018). Future directions in childhood adversity and youth psychopathology. Future Work in Clinical Child and Adolescent Psychology.

[38] Ertekin Z., Gunnar M.R., Berument S.K. (2021). Temperament moderates the effects of early deprivation on infant attention. Infancy.

[39] Gregory T., Dal Grande E., Brushe M., Engelhardt D., Luddy S., Guhn M., Brinkman S. (2021). Associations between school readiness and student wellbeing: A six-year follow up study. Child Indic. Res..

[40] Bakermans-Kranenburg M., IJzendoorn M., Juffer F. (2008). Earlier is better: A metaanalysis of 70 years of intervention improving cognitive development in 65 institutionalized children. Monogr. Soc. Res. Child Dev..

[41] Tetik N., Sen G. (2021). Impact of adolescents’ sleeping problems and habits on the quality of their sleep. J. Turk. Sleep Med..

[42] Kulari G., Pereira de Castro M. (2024). Depressive symptoms, loneliness and social support in healthcare employees: Does the source of support matter?. J. Public Ment. Health.

[43] Dunning D.L., Griffiths K., Kuyken W., Crane C., Foulkes L., Parker J., Dalgleish T. (2019). Research review: The effects of mindfulness-based interventions on cognition and mental health in children and adolescents—A meta-analysis of randomized controlled trials. J. Child Psychol. Psychiatry.

[44] Zelazo P.D., Lyons K.E. (2012). The potential benefits of mindfulness training in early childhood: A developmental social cognitive neuroscience perspective. Child Dev. Perspect..

[45] Brand S., Kirov R. (2011). Sleep and its importance in adolescence and in common adolescent somatic and psychiatric conditions. Int. J. Gen. Med..

[46] Figueiredo S. (2022). Sleeping Habits Explaining Academic Vulnerability and Household Influence: Co-sleeping and the Impact on Children’s Fluid Intelligence. Eur. J. Educ. Res..

